# Resource allocation in public sector programmes: does the value of a life differ between governmental departments?

**DOI:** 10.1186/s12962-023-00500-5

**Published:** 2023-12-15

**Authors:** Patricia Cubi-Molla, David Mott, Nadine Henderson, Bernarda Zamora, Mendel Grobler, Martina Garau

**Affiliations:** 1 Office of Health Economics, London, UK; 2 Department of Surgery and Cancer, Imperial College London, London, UK; 3 Medison Pharma, Sydney, Australia

**Keywords:** Resource allocation, Health, Public sector, Value of a QALY, Value of a life, Demand-side threshold, Supply-side threshold, Policy threshold, Decision rule, VSL, Willingness to pay, Opportunity cost

## Abstract

**Background:**

The value of a life is regularly monetised by government departments for informing resource allocation. Guidance documents indicate how economic evaluation should be conducted, often specifying precise values for different impacts. However, we find different values of life and health are used in analyses by departments within the same government despite commonality in desired outcomes. This creates potential inconsistencies in considering trade-offs within a broader public sector spending budget. We provide evidence to better inform the political process and to raise important issues in assessing the value of public expenditure across different sectors.

**Methods:**

Our document analysis identifies thresholds, explicitly or implicitly, as observed in government-related publications in the following public sectors: health, social care, transport, and environment. We include both demand-side and supply-side thresholds, understood as societies’ and governments’ willingness to pay for health gains. We look at key countries that introduced formal economic evaluation processes early on and have impacted other countries’ policy development: Australia, Canada, Japan, New Zealand, the Netherlands, and the United Kingdom. We also present a framework to consider how governments allocate resources across different public services.

**Results:**

Our analysis supports that identifying and describing the Value of a Life from disparate public sector activities in a manner that facilitates comparison is theoretically meaningful. The optimal allocation of resources across sectors depends on the relative position of benefits across different attributes, weighted by the social value that society puts on them. The value of a Quality-Adjusted Life Year is generally used as a demand-side threshold by Departments of transport and environment. It exceeds those used in health, often by a large enough proportion to be a multiple thereof. Decisions made across departments are generally based on an unspecified rationing rule.

**Conclusions:**

Comparing government expenditure across different public sector departments, in terms of the value of each department outcome, is not only possible but also desirable. It is essential for an optimal resource allocation to identify the relevant social attributes and to quantify the value of these attributes for each department.

## Background

Pressures on public sector budgets lead to concerns about the efficient allocation of public sector spending [1], along with searches for mechanisms to assess the ‘value for money’ of options considered [2]. Decisions on budget allocation between or across departments are generally made at a central political (Cabinet) level, but the factors, weights, and quantification of Cabinet level decisions are not readily publicly available. Possibly as a consequence of that, over recent decades, academic research effort has focussed on mechanisms to achieve a more efficient allocation of funds *within* specific departments or spending programmes [3–7].

In countries with a publicly funded universal healthcare system, there has been substantial discussion and academic debate about the efficiency of funding within the healthcare sector [8–10]. The opportunity cost (OC) of spending within health has been a focus of both policy and research. Much of the literature is dedicated to identifying “thresholds” above or below which the OC of spending is either acceptable or not. The methods used are, however, based on a core assumption that budgets are fixed [3, 11, 12]. Consequently, in healthcare, new projects, at least in theory, ‘compete’ for funding within a restricted budget for health, so that only those which are the most cost-effective should be funded [13–15].

In more pragmatic terms, countries around the world apply a form of health technology assessment (HTA) based on the consideration of an explicit or implicit cost-effectiveness threshold to recommend if new treatments should be funded for patients. The most common benchmarking metric used by HTA agencies is ‘cost per QALY’, where QALY stands for quality-adjusted life year. QALYs measure years of life, adjusted for quality of life linked to the health status during those years [16]. In England, for example, the National Institute for Health and Care Excellence (NICE) sets, for most technologies, £30,000 as the maximum cost to be incurred by the NHS to obtain an additional ‘unit of healthy life’, measured in terms of QALYs [17].

Historically, the measurement of quality of life has also been a research focus in a number of non-health sectors where health is an important metric in the outcome space, such as transport or the environment [18]. However, that research neither evolved into the development of a sector-specific quality-of-life related measure, nor facilitated the introduction of (already developed) QALYs as a generic outcome measure for those sectors. In non-health sectors, cost–benefit analysis is the most commonly used method to compare alternative interventions ‘competing’ for funding [19]. The assessment of benefits usually involves a monetary valuation of the outcomes produced by these interventions. In this context, the thresholds attain a different interpretation: they reflect the within-sector value provided to that outcome (typically addressed as societal ‘willingness to pay’ or WTP). WTP measures are generally used to rank projects according to their value for society (‘consumption value’ of health), and they are not usually sufficient as a rule for rationing: decision-makers still need to establish a *policy threshold* to ration and make the acceptance/rejection decisions [20]. For instance, health outcomes in England’s Department for Transport (DfT) are frequently assessed against benchmarks such as the value of a life or the value of a prevented fatality, used as WTP thresholds. However, not all projects for which the WTP (based on the value-of-a-life threshold) exceeds cost (i.e., for which the net present value estimated through the cost–benefit analysis is positive) are finally implemented. In reality, the actual rule used to decide on the implementation of the policy—i.e., the minimum net present value above which a policy will be implemented—is not made explicit in any form or shape.

Consequently, it is likely that the health-related outcomes of different interventions are measured and valued differently across public sector areas. We could think, for instance, of a policy that generates an amount of life gained which is considered as good value for money by one department but is rejected by another department that uses a different benchmarking threshold. For instance, the DfT in England is willing to pay up to £70K for a QALY gained [19], whereas the NICE threshold indicates that £20,000-£30,000 is sufficient to generate one QALY. These inconsistencies are a fundamental issue when considering trade-offs against a broader public sector spending budget. Recently, a number of authors have highlighted the importance of shifting the allocation framework and research towards an ‘all-encompassing’ multi-sector approach [2, 21–23]. This could begin to ameliorate these shortcomings.

While there is vast literature on the *social value of health* gains in specific sectors [8, 24–26], few researchers have sought to compare how health gains are appraised across public sectors [3, 27, 28]. Similarly, we identified in the literature a large number of papers on the empirical, *monetary valuation of health* from the OC perspective [29, 30], but these papers do not offer a pragmatic view of how much money is linked to an additional unit of comparable benefit (e.g. a QALY, a life year, a life) in different public sector departments or programmes. Finally, there is little research on identifying the (typically implicit) *policy threshold* that relates to the value of health that is considered when accepting or rejecting a policy with a direct impact on health. Note that a policy threshold may differ from the most accurate empirical estimate of OC (supply-side threshold) and from the social value of health (demand-side threshold). Explicit policy thresholds used in HTA arena are the clear exception, in the sense that, for some countries, the rationing rule is clearly described in policy documents (e.g., the £20,000-£30,000 NICE threshold); and yet, empirical retrospective analysis suggests that the actual policy threshold may considerably deviate from the ones described in the documents [9].

The aim of our paper is to assess if there are differences in the value given to health across country-specific departments; quantify these differences and identify the reasons underlying them; and explore the potential effects on policy decision making. For this purpose, we identify and compare estimates of the value of life and health used to inform resource allocation within three government departments: health, transport, and environment. We focus on these three departments for two reasons: improving health (or avoiding health losses) is an important part of their objectives; and, historically, the measurement of quality of life has been a research focus in these sectors [18]. Our search did not aim to focus on a particular type of threshold, but to identify all the valuations of health explicitly or implicitly used in government-related publications to represent the government’s value for money threshold, regardless of the approach followed to determine it (a discussion of the different approaches can be found elsewhere [31, 32]).

As suggested by several authors, in absence of any other prioritisation criteria, both the absence of a threshold and the use of thresholds that are too high, too low, or too many, risks an unfair and inefficient allocation of resources [13, 31]. In this sense, our research is intended to raise a number of important issues on assessing the value of public expenditure across different sectors and so better inform the policy process. We focussed on a group of countries that showed commitment to implement formal health economic evaluation early on [33, 34]; and/or introduced mandatory or recommended guidance on the conduct of health economic evaluation that has been revised subsequently, indicating an active use of economic evaluation [34]. The selected countries are: Australia, Canada, Japan, New Zealand, the Netherlands, and the United Kingdom (UK).

This paper is structured as follows. The next section explores whether it is theoretically meaningful to compare government expenditure across different public sector departments, in terms of the value of each departmental outcome. Section "Methods" introduces the measures for valuing health that are considered in this paper and provides the methods used to map across measures. The document search methodology and validation exercise are also detailed in this section. Section "Results" provides the results of the document analysis through inter-country comparison. Section "Discussion" provides a discussion of results and limitations, and section "Conclusions" concludes.

## A framework for comparing the value of public sector outcomes

In this section, we present a framework to describe how governments allocate resources across different public services. We begin with a model framed in the welfare economics literature, which provides the foundation for most approaches to allocative efficiency [35]. We take a Pareto optimality perspective and a utilitarian standpoint, which measures social welfare as the sum of individuals’ utility. In particular, our model builds on the resource-allocation model suggested by Meltzer and Smith [15] and Martin et al. [14]; it categorises the various policy outcomes into principal attributes (or outcome types), differentiating between health (H) and non-health (A) attributes. In relation to health attributes, we use the optimisation model frequently found in the health economics literature [3, 36–38].

**Fig. 1:**
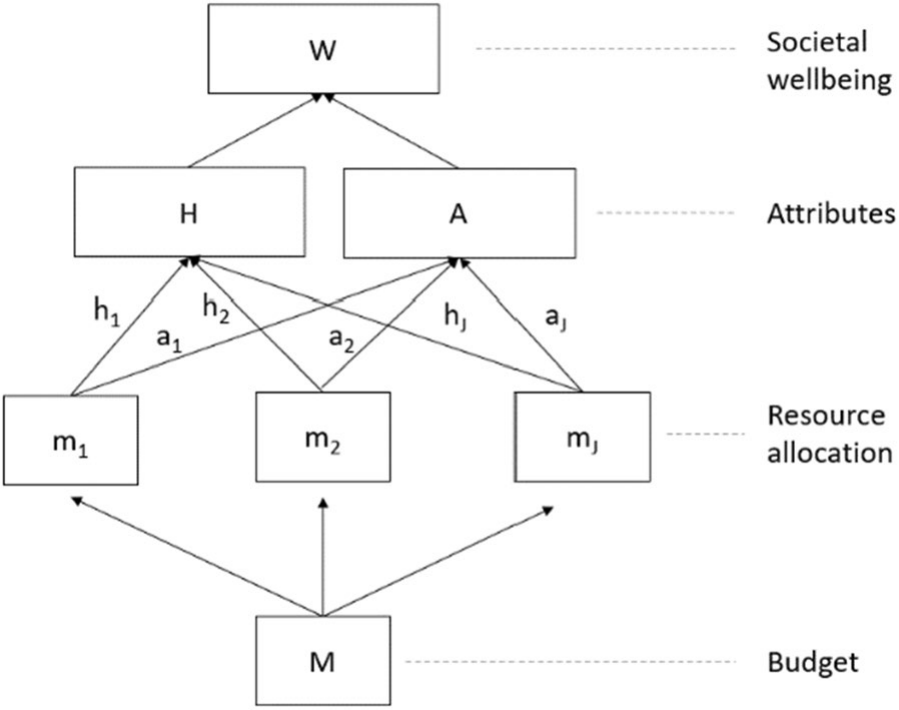
Elements of our theoretical model of resource allocation

The model is illustrated in Fig. 1. The government seeks to allocate its budget, *M* comprising $\left\{{m}_{1},{m}_{2},\dots ,{m}_{J}\right\}$, across the departments *j* = *{1,…,J}*, in such a way as to maximise the total welfare of society $W\left(H,A\right)$. Every government department invests ${m}_{j}$ to generate ${h}_{j}$ health outcomes and ${a}_{j}$ outcomes in non-health attributes. For consistency, we assume that all the outcomes ${h}_{j}$ and ${a}_{j}$ can be expressed in terms of monetary benefits.

In this simple model, the optimal allocation of budgets is achieved in a scenario where a marginal increase (e.g., one dollar or one pound) in the budget of any department will have the same total effect on the social welfare function, through health and non-health attributes. Details on the optimisation problem can be found in Appendix 1. We define **υ = **$\frac{\partial {\varvec{W}}}{\partial {\varvec{H}}}/\frac{\partial {\varvec{W}}}{\partial {\varvec{A}}}$ as the social value of health relative to the non-health attribute *A*. In a *W* maximising equilibrium, the following relationship for any two departments *i* and *j* needs to hold:

$$ \mathrm{\upsilon}\left(\frac{\partial {\text h}_{\text i}}{\partial {\text m}_{\text i}}-\frac{\partial {\text h}_{\text j}}{\partial {\text m}_{\text j}}\right)=\frac{\partial {\text a}_{\text j}}{\partial {\text m}_{\text j}}-\frac{\partial {\text a}_{\text i}}{\partial {\text m}_{\text i}}\text{ for each i, j} \, \text{in 1, 2,} { \ldots, \text{J}}$$

For illustrative purposes, consider two government departments: health and social care (*i* = HSC) and transport (*j* =  T); and two attributes: improved health (*H*) and improved standards of living (*A*). In this context, υ signals the relative value that improved health has for the society, in relation to improved standards of living. Note that this is consistent with the interpretation of a threshold as the societal value for health. In contrast, the health benefit generated by a marginal change in the HSC budget, $\frac{\partial {h}_{HSC}}{\partial {m}_{HSC}}$, is more aligned with the interpretation of thresholds in the sense of OC: they are reflecting the (inverse of the) ‘price’ in monetary units of purchasing one additional unit of health. We could observe that resources allocated to the transport sector can have a positive impact on health—yet may not be not as cost-effective in terms of health generation as investments in HSC sector $\left(\frac{\partial {h}_{HSC}}{\partial {m}_{HSC}}>\frac{\partial {h}_{T}}{\partial {m}_{T}}\right)$; however, the transport sector can possibly make a more efficient contribution to the improvement of non-health standards of living than HSC sector $\left(\frac{\partial {a}_{T}}{\partial {m}_{T}}>\frac{\partial {a}_{HSC}}{\partial {m}_{HSC}}\right)$. The optimal allocation of resources between both sectors depends on the relative position of differences of benefits in both attributes, weighted by the social value that society puts on health in relation to standards of living: $\mathrm{\upsilon}\left(\frac{\partial {h}_{HSC}}{\partial {m}_{HSC}}-\frac{\partial {h}_{T}}{\partial {m}_{T}}\right)=\frac{\partial {a}_{T}}{\partial {m}_{T}}-\frac{\partial {a}_{HSC}}{\partial {m}_{HSC}}$.

This paper identifies estimates of the different inputs that enter the equation above: societal values (WTP thresholds) which would determine the relative exchange rate (υ); and the decision-rules thresholds, which we assume that are based on the OC approach. In other words, we aim to identify the figures that relate to the monetary expenditure behind producing health at different public sector departments; and we do not explore the way that the monetary figure has been fixed, or the use that is made of it—either from supply-side or demand-side approaches, or even simply by using a convenience number based on past expenditure.

## Methods

### Measuring the value of life and health

The estimation of ‘value of life and health’ measures requires instruments aimed to capture the value of reducing the risk of death (mortality risks), increasing life expectancy, or improving health-related quality of life [27, 39].

There are several different approaches to representing the value of life and health. The value of a statistical life (VSL; also known as the value of a prevented fatality or VPF), is one of the most applied methods. Two approaches can be used to estimate a VSL, which are based on revealed preferences (e.g., the wage-risk approach, also referred to as the wage differential or labour market method) [27], or stated preferences (e.g., WTP or contingent valuation methods). The WTP approach involves asking a sample of participants to each state their WTP for a small risk reduction, which is then translated into an overall VSL estimate. Thus, as noted by Mason et al. [40], a WTP-based VSL can be defined as the aggregate WTP across a large group of individuals for small risk reductions. Importantly therefore, a WTP-based VSL does not represent the amount that an individual is willing to pay to save a life.

Another measure, useful for analyses of programmes or interventions that result in a small number of years of life being saved, is the value of a life year (VOLY; or statistical life year SLY). VOLYs can be derived directly (e.g., using a WTP approach), or indirectly from an existing VSL estimate [41]. The latter essentially involves dividing a population-level VSL by average life expectancy [40, 42].

In some cases, particularly in the health setting, it is important to consider both morbidity and mortality. That is, quality of life gains should be considered alongside survival gains. The quality-adjusted life year (QALY) achieves this by combining health state utilities (where 0 is equivalent to dead, and 1 is equivalent to full health) with survival gains, such that a year in full health corresponds to one QALY.

A clear unifying framework that demonstrates the conceptual link between VSLs, VOLYs, and willingness to pay for quality-adjusted life years (WTP-QALYs) has been provided in a recent study conducted on behalf of the Health and Safety Executive in the United Kingdom [39]. Future research may be able to utilise this framework to obtain value of life estimates that are better aligned and consistent. In lieu of such aligned estimates, comparisons between measures are inevitably imperfect.

In this paper, we will use the expression ‘VOQ’ to refer to every threshold benchmarking used when life and health are measured in terms of QALYs (VOQ = ‘value of QALY’). Both demand-side thresholds (referred to as ‘WTP-QALY’) and supply-side thresholds will be captured under that label. This is because our aim is not to distinguish between different types of thresholds; but to identify those explicitly or implicitly addressed in government-related publications, which represent the government’s value for money threshold in the context of health gains. Similarly, for simplification, we will use the expression ‘value of life’ or ‘VoL’ to encompass all types of estimates of the value of health and life (i.e., VSLs, VOLYs, and VOQs). This equivalence in the nomenclature is detailed in Table 1.

**Table 1:** Terminology for value of life estimates

	VoL Value of Life		
Measures	VSL *Value of a Statistical Life*	VOLY *Value of a Life Year*	VOQ *Value of a QALY*
Also known as	VPF *Value of a Prevented Fatality*	SLY *Statistical Life Year*	Social value of a QALY WTP-QALY Decision threshold Policy threshold
Main estimation approaches	Human Capital Wage-risk trade-off WTP	WTP	Supply-side constraints WTP

WTP: Willingness-to-pay; QALY: Quality-adjusted life year

In order to compare estimates of the VoL (Table 1) between governmental departments, it is necessary to convert them into equivalent metrics. Given the primary focus on health, we set out to compare figures in terms of VOQs. As VoL in transport and environmental settings is often presented as the value of a statistical life (VSL) or the value of a life year (VOLY) we use the formula from Abelson [27] to convert these from VSLs to VOLY estimates. The latter were subsequently converted to VOQ estimates, which required country-specific discount rates and life expectancy estimates, as detailed in Appendix 2.

We anticipated high sensitivity of the estimates to the various assumptions; therefore, we conduct sensitivity analyses to test the robustness of our findings. This includes varying the expected remaining life expectancy in the VSL/VOLY conversions (i.e., using ages other than 40) and varying the conversion rate between VOLYs and VOQs (with an upper limit of 1:1 based on the rationale outlined above).^1^

### Document analysis

The aim of the document search and analysis was to identify whether VoL thresholds exist within the health, social care, transport, and environment departments of the six selected countries and to determine which thresholds are used to inform resource allocation decisions. In the case of Health Departments, we aimed to identify whether there was any variation in the VoL estimates used within the department, for example, thresholds used in HTA evaluation compared to public health. For the other departments, we sought to identify the main VoL indicators, and we did not seek to explore if other thresholds were used in sub-programmes, or the use of these thresholds in impact assessment documents.

The document search gathered evidence from technical reports, guidelines, and tools published directly by government departments in each of these countries indicating methods for conducting impact assessments (e.g. Green Book in the UK [19, 43]), where necessary other published literature was explored.

Sources were identified using two methods. Firstly, a targeted review of government and departmental websites was conducted, seeking to identify official guidelines and tools published for use in impact assessments or cost–benefit analysis and so on. We identified the most recent estimate available. Secondly, EconLit database was searched, using variations of the terms “Value of life” and “Government/Department” and “UK/Australia/New Zealand/…”. Only documents in English were included. Papers were deemed relevant if they reported an explicit or implicit value of life or threshold in any department under consideration. In both cases, documents were prioritised if they stated an explicit VoL threshold. For countries where no explicit threshold exists or could be identified, implicit thresholds e.g., thresholds elicited in academic papers and cited as relevant in official documents were accepted as the best proxy available. Further details of the document search and data gathering can be found elsewhere [44].

To make comparisons across departments, we estimated VoL in transport and environment departments as a proportion of the VoL in the health setting. These proportions could then be compared across countries to provide a clear indication of whether the same trends are found between countries and without having to consider variations in exchange rates or the need for purchasing power parity.

## Results

An estimate of value of life or health was suitably identified for all departments and for all countries. Generally, it was not possible to distinguish between guidance for health and for social care. Therefore, we focused solely on health.

Most of the thresholds identified in the search related to demand-side thresholds or policy thresholds. The Department of Health and Social Care (DHSC) in the UK provided the only exception, as it appears to have recently adopted an explicit supply-side threshold of £15,000 of additional spending per QALY gain for projects excluding HTA.^2^ This figure, used in impact assessments [45, 46], is based on an estimate from Claxton et al. [30]. The adoption of £15,000 as an explicit supply-side threshold was also discussed at the review of Cost-Effectiveness Methodology for Immunisation Programmes & Procurements [47]; but the adoption of this figure was finally rejected by the government [48]. Our search did not identify any explicit, evidence-based supply-side thresholds elsewhere. Therefore, this section primarily focuses on our findings on demand-side and policy thresholds.

To maximise comparability, values have been adjusted to 2019 prices wherever possible. All prices are shown in the local currency (i.e., GBP, EUR, CAD, JPY, AUD & NZD). Tables 2, 3, 4, 5, 6, 7 show the estimates identified for the UK, the Netherlands, Canada, Japan, Australia, and New Zealand.

**Table 2:** Value of life in the UK

No	Area	Source	Relevance	Type of estimate	Stated value (£)	Year	2019/2020 Value* (£)	VOLY (£)	VSL (£)	QALY (£)
1	Health	NICE	Threshold used to assess ‘normal’ treatments	QALY	20,000–30,000	2013	20,000–30,000	18,399–27,599	663,351–995,027	**20,000–30,000**
2	Health	NICE	Threshold used to assess ‘end-of-life’ treatments****	QALY	50,000	2013	50,000	45,998	1,658,378–3,316,756	**50,000**
3	Health	NICE	Threshold used to assess ‘highly specialised technologies’	QALY	100,000–300,000	2017	100,000–300,000	91,996–275,988	3,316,756–9,950,268	**100,000–300,000**
4	Health	HM Treasury	Health-related appraisal (Green Book)	QALY	60,000	2018	60,000	55,198	1,990,054	**60,000**
5	Health	HM Treasury	Health-related appraisal (Green Book)	QALY	70,000**	2022	70,000	64,397	2,321,729	**70,000**
6	Health	DHSC	Adopted in relevant Impact Assessments	QALY	15,000***	2016	15,000	13,799	497,513	**15,000**
7	Transport	HM Treasury/DfT	Social cost–benefit analysis (Green Book)	VPF	2,064,189	2020	2,064,189	67,650	**2,064,189**	73,535
8	Environment	Defra	Valuing life lost due to chronic effects of air pollution	VOLY	42,780	2017	*45,343*	**45,343**	1,383,543	49,288
9	Environment	Defra	Valuing life lost due to acute effects of air pollution	VOLY	22,110	2017	*23,435*	**23,435**	715,068	25,474
10	Other	HM Treasury	Social cost–benefit analysis (Green Book)	SLY	60,000	2018	60,000	**60,000**	1,830,770	65,220

*Values were only updated to 2019/2020 values if they were not already up to date, and if guidance suggests that this is required/appropriate. Only the values in italics were adjusted. Values in bold are the original values, after adjustments. A discount rate of 1.5% and life expectancy of 81.1 were used in calculations. All values are in local currency

**This reference was introduced after the conclusion of our document search and analysis. As it is an inflationary adjustment of the threshold No. 4, this figure has not been included in the analysis

***We could not identify an official document recommending the use of this supply-side threshold—beyond its use in impact assessment reports. We include the figure here for completeness. However, since impact assessment reports were beyond the scope of our search for other countries, we do not include this threshold in our analysis, as a matter of consistency

****This has nowadays been replaced by a severity adjustment that has the same budget impact as a £50,000 threshold

**Table 3:** Value of life in the Netherlands

No	Area	Source	Relevance	Type of estimate	Stated value (€)	Year	2019/2020 Value* (€)	VOLY (€)	VSL (€)	QALY (€)
1	Health	ZIN	Treatments with proportional QALY shortfall of 0.10–0.40	QALY	20,000	2015	20,000	18,399	671,639	**20,000**
2	Health	ZIN	Treatments with proportional QALY shortfall of 0.41–0.70	QALY	50,000	2015	50,000	45,998	1,679,098	**50,000**
3	Health	ZIN	Treatments with proportional QALY shortfall of 0.71–1.00	QALY	80,000	2015	80,000	73,597	2,686,557	**80,000**
4	Transport	OEEI Guideline	Values for transport infrastructure projects: European average from an OECD publication	VPF	1,500,000	1998	1,500,000	48,553	**1,500,000**	52,777
5	Environment	CE Delft	Valuing life lost due to air pollution (though VOLY is preferred)	VSL	2,400,000	2012	2,400,000	77,685	**2,400,000**	84,443
6	Environment	CE Delft	Valuing the health impact of environmental pollution	VOLY	70,000	2015	*74,244*	**74,244**	2,293,699	80,703
7	Other	SEO	Values for social cost–benefit analysis in the social domain	QALY	50,000–100,000	2015	50,000–100,000	45,998–91,996	1,679,098–3,358,196	**50,000–100,000**
8	Other	CE Delft	Central estimate of the range for social cost–benefit analysis in the social domain	QALY	70,000	2015	70,000	64,397	2,350,737	**70,000**

*Values were only updated to 2019/2020 values if they were not already up to date, and if guidance suggests that this is required/appropriate. Only the values in italics were adjusted. Values in bold are the original values, after adjustments. A discount rate of 1.5% and life expectancy of 81.1 were used in calculations. All values are in local currency

**Table 4:** Value of life in Canada

No	Area	Source	Relevance	Type of estimate	Stated value (CA$)	Year	2019/2020 Value* (CA$)	VOLY (CA$)	VSL (CA$)	QALY (CA$)
1	Health	PMPRB	Estimate of the lower bound of the QALY threshold range	QALY	50,000	2019	50,000	45,998	1,683,060	**50,000**
2	Health	PMPRB	Estimate of the upper bound of the QALY threshold range (oncology drugs)	QALY	100,000	2019	100,000	91,996	3,366,119	**100,000**
3	Health	Skedgel et al	Academic estimate of an implied threshold for pCODR (oncology drugs)	QALY	140,000	2018	140,000	128,794	4,712,567	**140,000**
4	Health	PMPRB	Proposed future threshold value	QALY	60,000	2020	60,000	55,198	2,019,672	**60,000**
5	Transport	TBS	Estimate for use in cost–benefit analysis	VSL	6,110,000	2004	*8,079,656*	260,912	**8,079,656**	283,612
6	Environment	TBS	Estimate for use in cost–benefit analysis	VSL	6,110,000	2004	*8,079,656*	260,912	**8,079,656**	283,612

*Values were only updated to 2019/2020 values if they were not already up to date, and if guidance suggests that this is required/appropriate. Only the values in italics were adjusted. Values in bold are the original values, after adjustments. A discount rate of 1.5% and life expectancy of 81.1 were used in calculations. All values are in local currency

**Table 5:** Value of life in Japan

No	Area	Source	Relevance	Type of estimate	Stated value (¥)	Year	2019/2020 Value* (¥)	VOLY (¥)	VSL (¥)	QALY (¥)
1	Health	MHLW	Price adjustment threshold for ‘normal’ treatments	QALY	5,000,000–10,000,000	2019	5,000,000–10,000,000	4,599,800–9,199,600	158,701,235–317,402,469	**5,000,000–10,000,000**
2	Health	MHLW	Price adjustment threshold for products with ‘special considerations’	QALY	7,500,000–15,000,000	2019	7,500,000–15,000,000	6,899,700 13,799,400	238,051,852 476,103,704	**7,500,000–15,000,000**
3	Transport	Cabinet Office Morisugi et al	Estimates in relation to road accidents	VSL	226,000,000–462,000,000	2007	226,000,000–462,000,000	7,739,790–15,822,049	**226,000,000–462,000,000**	8,413,152–17,198,567
4	Environment	Itaeka et al	Estimates in relation to air pollution	VSL	103,000,000–344,000,000	1999	103,000,000–344,000,000	3,527,427–11,780,920	**103,000,000–344,000,000**	3,834,313–12,805,860

*Values were only updated to 2019/2020 values if they were not already up to date, and if guidance suggests that this is required/appropriate. Only the values in italics were adjusted. Values in bold are the original values, after adjustments. A discount rate of 1.5% and life expectancy of 81.1 were used in calculations. All values are in local currency

**Table 6:** Value of life in Australia

No	Area	Source	Relevance	Type of estimate	Stated value (AU$)	Year	2019/2020 Value* (AU$)	VOLY (AU$)	VSL (AU$)	QALY (AU$)
1	Health	Henry, Hill & Harris	Estimate based on PBAC decisions between 1994–2003	QALY	52,400	2003	52,400	48,206	996,295	**52,400**
2	Health	Paris & Belloni	Estimate of maximum cut-off based on PBAC decisions between 2005–2009	QALY	75,000	2009	75,000	68,997	1,425,994	**75,000**
3	Health	Wang, Gum & Merlin	Threshold used to compare PBAC and NICE decisions 2005–2015	QALY	50,000	2015	50,000	45,998	950,663	50,000
4	Transport	BITRE	Estimate of the cost per road fatality	VSL	2,400,000	2006	*3,215,595*	183,838	**3,215,595**	199,832
5	Environment	NEPC/Boulter & Kulkarni	Economic analysis to inform the National Plan for Clean Air	VOLY	288,991	2011	288,991	**288,991**	5,054,867	314,133
6	Environment	NEPC/Boulter & Kulkarni	Economic analysis to inform the National Plan for Clean Air	VSL	6,000,000	2006	6,000,000	343,025	**6,000,000**	372,868
7	Other	PM&C	For use in cost–benefit analyses for regulation impact statements	VOLY	213,000	2019	213,000	**213,000**	3,725,676	231,531
8	Other	PM&C	For use in cost–benefit analyses for regulation impact statements	VSL	4,900,000	2019	4,900,000	280,137	**4,900,000**	304,509

*Values were only updated to 2019/2020 values if they were not already up to date, and if guidance suggests that this is required/appropriate. Only the values in italics were adjusted. Values in bold are the original values, after adjustments. A discount rate of 1.5% and life expectancy of 81.1 were used in calculations. All values are in local currency

**Table 7:** Value of life in New Zealand

No	Area	Source	Relevance	Type of estimate	Stated value (NZ$)	Year	2019/2020 Value* (NZ$)	VOLY (NZ$)	VSL (NZ$)	QALY (NZ$)
1	Health	Pritchard et al	Academic estimate based on PHARMAC decisions from 1998–2001	QALY	20,000	2001	*28,342*	26,073	667,896	**28,341**
2	Health	Treasury	Value from the CBAx tool for use in cost benefit analysis	QALY	33,306	2019	33,306	30,640	784,904	**33,306**
3	Transport	Ministry of Transport	Estimate in the context of road crashes and injuries	VSL	4,340,000	2018	*4,494,540*	207,311	**4,494,540**	225,347
4	Transport	Treasury	Value from the CBAx tool for use in cost benefit analysis	VSL	4,900,000	2019	4,900,000	226,013	**4,900,000**	245,676
5	Environment	Treasury	Value from the CBAx tool for use in cost benefit analysis	VSL	4,900,000	2019	4,900,000	226,013	**4,900,000**	245,676

*Values were only updated to 2019/2020 values if they were not already up to date, and if guidance suggests that this is required/appropriate. Only the values in italics were adjusted. Values in bold are the original values, after adjustments. A discount rate of 1.5% and life expectancy of 81.1 were used in calculations. All values are in local currency

Table 8 illustrates that in 13 out of 15 comparisons the values in the health setting are far lower than those from the transport and environment settings, with the value of life used in health often < 50% of the value used in transport (in six out of seven comparisons) and environment (in five out of eight comparisons). There are only two examples where the value in transport or environment does not exceed the value used in health. The first is the smaller of the two Defra estimates in the UK, which is intended for capturing the acute mortality impact of air pollution. If a broader estimate had been used, such as one from the Green Book, the value would be at least double the upper health value of £30,000 (see Table 2). The other example is the smaller of the two academic estimates in Japan, also in an environment setting, which was the lowest value from that particular study.

**Table 8:** Inter-country comparison

Country	Health (HTA)		Transport			Environment		
	Source	Value(s)	Source	Value	Health as %	Source	Value	Health as %
UK	NICE	20,000–30,000	DfT/Green Book	73,535	27–41%	Defra	25,474	79–118%
							49,288	41–61%
NL	ZIN	20,000	OEEI Guideline	52,777	38%	CE Delft	80,703	25%
CA	PMPRB	50,000–100,000	Treasury	283,612	18–35%	Treasury	283,612	18–35%
JP	*Implicit*	5,000,000	Cabinet Office	8,413,152	59%	Academic	3,834,313	130%
				17,198,567	29%		12,805,860	39%
AU	Academic (× 2)	52,400–75,000	BITRE	199,832	26–38%	NEPC	314,133	17–24%
NZ	Treasury	33,306	Treasury	245,676	14%	Treasury	245,676	14%

All values are in local currency. *: England. HTA: Health Technology Assessment. UK: United Kingdom. NL: The Netherlands. CA: Canada. JP: Japan. AU: Australia. NZ: New Zealand. NICE: National Institute of Health and Care Excellence. ZIN: Zorginstituut Nederland (National Health Care Institute). PMPRB: Patented Medicine Prices Review Board. DfT: Department for Transport. BITRE: Bureau of Infrastructure and Transport Research Economics. Defra: Department for Environment, Food and Rural Affairs. NEPC: National Environment Protection Council

**Fig. 2:**
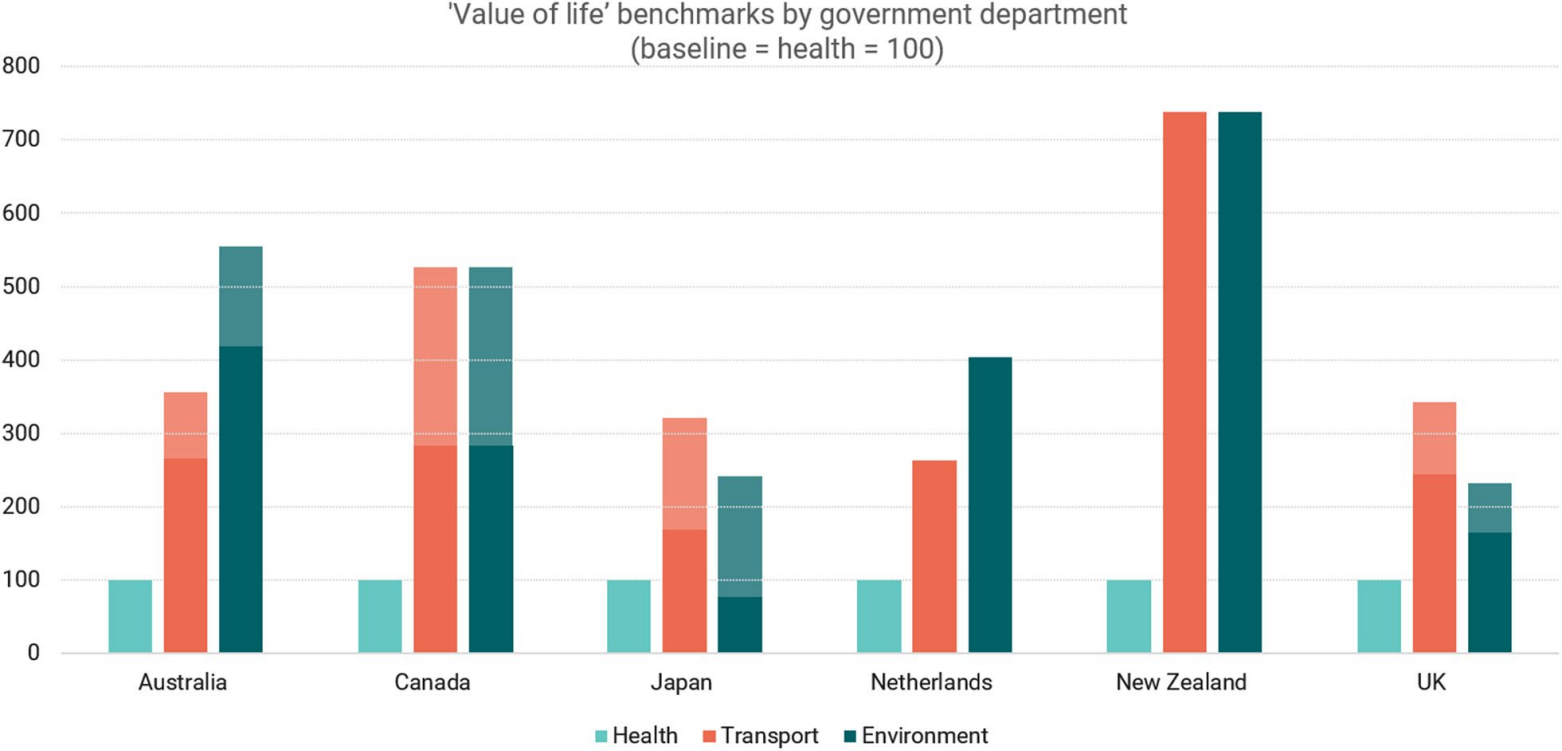
‘Value of life’ benchmarks by government department. Health = baseline (100)

Figure 2 presents our findings graphically. Using each country’s value of life estimate from the health setting as the baseline, we show transport and environment estimates relative to health. The shaded areas indicate the range of the estimates. The largest difference between value of life estimates in the health setting compared to transport and environment is seen in New Zealand, but there are sizeable differences in all countries considered.

The sensitivity analysis found that the observed trend that health is valued less in the health setting relative to transport and environment setting appears to be robust to the assumptions made when converting between measures. Further details of the sensitivity analysis can be found in Appendix 3.

## Discussion

Our research has two main contributions to the literature on resource allocation. First, we provide a simple model on resource allocation that is consistent with two interpretations of the threshold: as the societal value for health (the relative value that improved health has for the society, in relation to other non-health attributes), and the opportunity cost (in the sense of the ‘price’ in monetary units of purchasing one additional unit of health). This theoretical framework supports that the optimal allocation of resources between both sectors depends on the relative position of differences of benefits between health and other attributes, weighted by the social value that society puts on health in relation to other attributes. Second, we examine the policy-related documents in three public sector areas, identify the main VoL estimates used within each department, and make comparisons between them.

From a theoretical perspective, as we have set out in the framework in section "A framework for comparing the value of public sector outcomes", if we assume that the ultimate goal of governments when distributing resources is to make optimal decisions for their citizens, then the methods used for economic evaluations of any government investment must be consistent, and resources should be allocated efficiently across and within departmental portfolios. It is important to recognise that a measure for use within health systems prioritisation (which is what the QALY provides) does not necessarily translate to overall resource allocation. The current cost per QALY threshold is used in a number of countries to determine if new technologies should be introduced into the HSC sector given the current budget constraint. On that basis, the threshold needs to be set at a level which reflects the OC in terms of health gain within the existing health budget. This, of course, raises questions of whether the health budget is optimal and how a government should determine the OC of putting additional resources into the health system or environment or transport, or any other department.

Our analysis also supports the fact that identifying and describing the VoL from disparate public sector activities, in a manner that facilitates comparison, is theoretically meaningful. First, benefits will be assessed around a set of attributes or domains that are relevant to society—‘health’ being one of them. This assumption is supported by a large number of papers that seek to identify country-specific public sector outcome attributes. For pragmatic reasons, ‘health and life’ benefits from different areas of public spending are usually described and measured in a variety of different units (as QALYs, VOLYs, or VSL), so it is typically very challenging to compare the value and OC of new investments. However, under a set of assumptions, it is plausible to estimate equivalences between these outcome measures, as long as a posterior sensitivity analysis proves that results are robust to changes in the assumptions supporting the mapping.

Fully variable budgets are probably the most argued condition for the social welfare model suggested by this report. The alternative, in which investments are made within an exogenously determined, annually ‘fixed’ budget, risks decisions being made that are suboptimal in the present and in the future. To move to an optimal distribution, criteria for approval should attract additional resources as long as the total increase in the welfare resulting from improvement in the ‘best’ attributes outperform the welfare lost resulting in a lower improvement in the ‘worst’ attributes relative to the other departments. Economic evaluations should take into account all costs and benefits that are important to society [49, 50] to facilitate the comparison of attributes across sectors; yet, societal preferences in defining the value of the attributes (in terms of trade-offs) are needed to correct the imbalances across sectors [22]. Finally, it is important to notice that when considering efficient allocation across sectors, we need an exchange rate based on societal values; and as it is an exchange rate (as illustrated in our theoretical model), it has to be based on the relative value that improved health has for the society, in relation to other (non-health) attributes.

More pragmatically, there is evidence in the countries studied that the VoL criteria used by those in the health sector are systematically lower than that those used for health gain achieved in comparable sectors. In some countries, this is considerably lower than the VoL provided in other non-health departments. In the vast majority of cases, VoL in the health setting is far lower than VoL in the transport and environment settings.

Empirically, we need to correlate some of these differences in VoL with the fact that transport and environment departments (as well as HSC departments except for HTA-based decision-making) usually use cost–benefit analysis as a form of economic evaluation to inform decision making. This advice translates into not using the threshold as a rationing rule. WTP measures are used to rank projects according to their value for society; but even if so, decision-makers need to establish a policy threshold related to the maximum value given to life and health, in order to ration and make the acceptance/rejection decisions in policies for transport, environment, and non-HTA health-related policies. Guidebooks identified in this paper do not explicitly state any policy threshold or rule to be used for that. Decisions made across departments such as transport or environment are thus generally based on an unspecified rationing rule which, in the absence of any other prioritisation criteria, risks an inefficient allocation of resources in the sense of overpaying for some new programmes. Whilst WTP measures thus overstate the actual policy threshold used for adopting new interventions in non-health departments it remains likely that the effective threshold for health gain is above the OC threshold used in health departments.

### Limits

Our study has a number of limitations. The first limitation relates to our ability to identify reliable estimates via a document search and analysis. We sought to identify the value of health estimates that are currently used in practice in analyses across multiple departments in multiple countries. The ideal sources for this information are governmental guidelines for the implementation of cost–benefit analyses, impact assessments or similar, such as the Green Book in the UK. Not all countries have an equivalent document, and, in some cases, such as in the Netherlands, guidelines existed, but explicit values of health were not provided. This meant that we often had to rely on other documentation that typically did not clearly state that a certain value is recommended or required to be used in analyses.

The second relates to our analysis. Whilst converting between VSLs and VOLYs is fairly well established, this is not the case for converting between VOLYs and QALYs. We identified a ratio in the literature and used this for our main analyses. However, it is not clear whether this is entirely appropriate. We tried to mitigate any impact of this by conducting a sensitivity analysis whereby the two measures were equivalent, as this would provide the smallest possible QALY values on the basis that a VOLY would not be valued more highly than a QALY.

A final limitation of the study is the lack of information found about the rationing (supply-side) rules used when non-health departments decide which projects should be implemented. These figures would allow a more accurate comparison of the value of life and health across public sectors, by considering supply- and decision- side rules separately.

### Future research

An exploration of the Impact Assessment reports could bring light to the unobserved policy threshold used for decision-making in non-health departments. Future research could also elicit the societal preferences for different attributes (and an exchange rate based on societal values subsequently), following an attribute framework suggested elsewhere [22]. With that information in hand, the optimal resource allocation suggested in this research could help quantify the potential imbalance in the value of health across different parts of the public sector.

## Conclusions

Comparing government expenditure across different public sector departments, in terms of the value of each department outcome, is not only possible but also desirable. In that respect, it is essential to identify the relevant social attributes and to quantify the value of the attributes for each public sector. We have shown that health is likely to be valued less by those responsible for allocating resources in health than those in the environment and transport portfolios. If decisions made across departments such as transport or environment are generally based on an unspecified rationing rule, risks of an inefficient allocation of resources in the sense of overpaying for some new programmes will always be present.

## Data Availability

Available on request.

## Appendices

### Appendix 1: Theoretical model

We present a model framed in the welfare economics literature, which provides the foundation for most approaches to allocative efficiency [35]. We take a Pareto optimality perspective and a utilitarian standpoint, which measures social welfare as the sum of individuals’ utility. We categorise the various policy outcomes into principal attributes (or outcome types), differentiating between health (H) and non-health (A) attributes. In relation to health attributes, we use the optimisation model frequently found in the health economics literature [3, 36–38].

The government seeks to allocate its budget, M comprising $\left\{{m}_{1},{m}_{2},\dots ,{m}_{J}\right\}$, across the departments *j* = *{1,…,J}*, in such a way as to maximise the total welfare of society $W\left(H,A\right)$. Every government department invests ${m}_{j}$ to generate ${h}_{j}$ health outcomes and ${a}_{j}$ outcomes in non-health attributes. For consistency, we assume that all the outcomes ${h}_{j}$ and ${a}_{j}$ can be expressed in terms of monetary benefits.

$$\left\{\begin{array}{c}\begin{array}{cc}Max& W(H,A)\\ s.t.& H={\sum }_{j}{h}_{j}\\ & A={\sum }_{j}{a}_{j}\end{array}\\ \begin{array}{cc}& {h}_{j}={f}_{j}\left({m}_{j},{z}_{j}\right)\\ & {a}_{j}={g}_{j}\left({m}_{j},{z}_{j}\right)\\ & {\sum }_{j}{m}_{j}\le M\end{array}\end{array}\right\}$$

Note that in our model of welfare-maximising decision-making, for simplicity we assume the government is acting as a perfect agent for society and has access to perfect information on the costs and benefits of extant and potential new public sector activities.

Also note that we are assuming a functional form for *H,* implying that $\partial W/\partial {h}_{j}$ is the same for all *j;* this translates into assuming that the output production process is irrelevant to its impact on the welfare function. For example, scenarios as societal preferences putting a higher value on producing health through education rather than through healthcare settings are not reflected in our model. However, this is not a necessary consideration in our work, given that it is the assessment of consistency in output valuation that is the goal.

The optimal level of expenditure in sector *j* derived from the model would be a function of the budget and the additional factors, as:

$${{m}_{j}^{*}=P}_{j}\left(M,{z}_{j}\right), j=1\ldots, J$$

In this simple model, the optimal allocation of budgets $\left\{{m}_{j}^{*}\right\}$ is achieved in a scenario where a marginal increase (e.g., one dollar or one pound) in the budget of any department ($\partial {m}_{j}$) will have the same total effect (‘marginal value’ or *MV*) in the social welfare function, through health and non-health attributes:

$$ {\text {MV}}_{\text i}=\frac{\partial \text W}{\partial \text H}\frac{\partial {\text h}_{\text i}}{\partial {\text m}_{\text i}}+\frac{\partial \text W}{\partial \text A}\frac{\partial {\text a}_{\text i}}{\partial {\text m}_{\text i}}=\frac{\partial \text W}{\partial \text H}\frac{\partial {\text h}_{\text j}}{\partial {\text m}_{\text j}}+\frac{\partial \text W}{\partial \text A}\frac{\partial {\text a}_{\text j}}{\partial {\text m}_{\text j}}={\text {MV}}_{\text j},\text{ for each i, j} \text{ in 1, 2,} { \ldots, }{\text{J}}$$

Following the same notation as in Claxton et al. (2010), we define υ = $\frac{\partial W}{\partial H}/\frac{\partial W}{\partial A}$ as the social value of health consumption relative to the non-health attribute *A*. The equilibrium formula leads us to:

$$\mathrm{\upsilon}\left(\frac{\partial {h}_{i}}{\partial {m}_{i}}-\frac{\partial {h}_{j}}{\partial {m}_{j}}\right)=\frac{\partial {a}_{j}}{\partial {m}_{j}}-\frac{\partial {a}_{i}}{\partial {m}_{i}}\text{, for each }i, j\text{ in } {1, 2,\ldots, {J}}$$

In situations where the *MV* of activities in one department exceeds that of another, available budgets shall be directed to the department with the higher *MV*. In principle, budget reallocation will occur until the *MV* is equal across all government departments—at that point, there is efficiency in public spending, social welfare is maximised, and any further reallocation of budgets would reduce social welfare.^3^

### Appendix 2: Mapping variables

In order to compare estimates of the VoL between governmental departments, it was necessary to convert them. Given the primary focus on health, we set out to compare figures in terms of VOQs, while we note that the VoL in transport and environmental settings is often presented as the value of a statistical life (VSL) or the value of a life year (VOLY).

Equation 1 sets out the formula used to convert from VSL to VOLY, from Abelson [27]:

1 $$\begin{array}{c}VOLY= \frac{\widehat{VSL}}{A}\end{array}$$

It therefore follows that the VSL can be calculated from a VOLY estimate:

2 $$\begin{array}{c}VSL= \widehat{VOLY}\cdot A\end{array}$$

A is defined as in Eq. 2:

3 $$\begin{array}{c}A=\frac{\left[1-{\left(1+r\right)}^{-n}\right]}{r}\end{array}$$

where* n* is the years of expected lifetime remaining and *r* is the rate at which future utility is discounted. The life expectancy and discount rate used in this calculation can have a significant impact on the overall estimates. For life expectancy, we followed convention by using life expectancy at age 40 [27]. Average life expectancy was identified for each country and 40 was subtracted from this figure to produce an estimate of *n* for each country. In practice, the most suitable age to calculate life expectancy from depends on the specific framing of the task used to estimate each VSL. Given the number of VSLs that we expected to identify and convert in this study, and the potential challenges associated with identifying the source of the numbers used in policy documents, we chose to use life expectancy at age 40 for all conversions. The implications of this assumption were explored in sensitivity analyses. For discount rates, government guidelines in each country were reviewed to identify suitable rates.

Converting VSL or VOLY to VOQ is challenging because the latter captures morbidity as well as mortality. Logically, it would be expected that QALY would be valued more highly than a life year, because it represents a year in full health as opposed to a year in average quality of life. The relationship between the two measures can be expressed as the following:

4 $$\begin{array}{c}{\text{V}}{\text{O}}{\text{Q}}={w}_{t}VOLY\end{array}$$

where *w*_*t*_ represents the proportional change in WTP as a result of moving from average quality of life (as experienced in one VOLY) to full health (as is experienced in one QALY). Thus, one VOLY is only equivalent to one QALY if there is no change in societal value when moving from average quality of life to full health.

Mason et al. and Tehard et al. both set out approaches to estimate VOQ from a VSL/VOLY [40, 42]. These approaches require comprehensive health state utility and life expectancy data. Their results indicate that WTP-QALYs are greater than VOLYs, in the region of 17–27% (a value of *w*_*t*_ of 1.17–1.27). This is considerably larger than the estimate of 8.7% cited in the CE Delft Environmental Prices Handbook [51]. Given the substantive data requirements for replicating the approaches by Mason et al. [40] and Tehard et al. [42], we instead decided to adopt the most conservative conversion rate that we identified in the literature (a value of *w*_*t*_ of 1.087) in our baseline comparisons and to conduct sensitivity analyses around this number.

Given the high sensitivity of the estimates to the various assumptions described above, we also conducted sensitivity analyses to test the robustness of our conclusions. This included varying the expected remaining life expectancy in the VSL/VOLY conversions (i.e., using ages other than 40) and varying the conversion rate between VOLYs and VOQs (with an upper limit of 1:1 based on the rationale outlined above).

### Appendix 3: Sensitivity analysis

As numerous assumptions are required to convert between VSLs, VOLYs and VOQs, it is important to test whether these assumptions could impact the findings. As noted earlier, two big assumptions relate to the age upon which life expectancy is calculated when converting between VOLYs and VSLs (40 was used in our analysis), and the conversion rate between QALYs and VOLYs (a rate of VOQ = 1.087 VOLY was used in our analysis). Both of these assumptions have clear limits. Using life expectancy at birth (age zero) will result in the smallest possible VOLYs when converting from VSLs, holding all else equal. Using a conversion rate of 1 to 1 between VOQs and VOLYs will result in the smallest possible VOQ estimates when converting from VOLY to VOQ. We therefore repeated the inter country comparison analysis using both of these alternative assumptions at the same time; the results are in Table 9.

**Table 9:** Sensitivity analysis results

Country	Health		Transport			Environment		
	Source	Value(s)	Source	Value	Health as %	Source	Value	Health as %
UK	NICE	20,000–30,000	DfT/Green Book	44,167	45–68%	Defra	23,435	85–128%
							45,343	44–66%
NL	ZIN	20,000	OEEI Guideline	31,951	63%	CE Delft	74,244	27%
CA	PMPRB	50,000–100,000	Treasury	171,956	29–58%	Treasury	171,956	29–58%
JP	Implicit	5,000,000	Cabinet Office	5,569,278	90%	Academic	2,538,211	197%
				12,375,477	40%		8,477,130	59%
AUS	Academic (× 2)	52,400–75,000	BITRE	163,696	32–46%	NEPC	288,991	18–26%
NZ	Treasury	33,306	Treasury	182,625	18%	Treasury	182,625	18%

Overall, the sensitivity analysis finds that the observed trend that health is valued less in the health setting relative to transport and environment setting appears to be robust to the assumptions made when converting between measures.

## Abbreviations

DfT: Department for Transport
HTA: Health Technology Assessment
HSC: Health and Social Care
OC: Opportunity cost
QALY: Quality-adjusted life years
VOL: Value of a life
VOLY: Value of a life year
VOQ: Value of a QALY
VSL: Value of a statistical life
WTP: Willingness to pay

## Footnotes

1 Excel files showing the sensitivity analysis are available upon request.

2 We could not identify an official document recommending the use of this supply-side threshold – beyond its use in impact assessment reports. Note that the £15,000 number is not used by the NHS in reviewing its spending programmes, or by NICE in its HTA appraisals on behalf of the NHS. We include the figure in the results for UK for completeness. However, since impact assessment reports were beyond the scope of our search for other countries, we do not include this threshold in our analysis, as a matter of consistency.
